# Enrichment and Stratification for Predementia Alzheimer Disease Clinical Trials

**DOI:** 10.1371/journal.pone.0047739

**Published:** 2012-10-17

**Authors:** Dominic Holland, Linda K. McEvoy, Rahul S. Desikan, Anders M. Dale, for the Alzheimer’s Disease Neuroimaging Initiative

**Affiliations:** 1 Department of Neurosciences, University of California San Diego, La Jolla, California, United States of America; 2 Department of Radiology, University of California San Diego, La Jolla, California, United States of America; Cardiff University, United Kingdom

## Abstract

The tau and amyloid pathobiological processes underlying Alzheimer disease (AD) progresses slowly over periods of decades before clinical manifestation as mild cognitive impairment (MCI), then more rapidly to dementia, and eventually to end-stage organ failure. The failure of clinical trials of candidate disease modifying therapies to slow disease progression in patients already diagnosed with early AD has led to increased interest in exploring the possibility of early intervention and prevention trials, targeting MCI and cognitively healthy (HC) populations. Here, we stratify MCI individuals based on cerebrospinal fluid (CSF) biomarkers and structural atrophy risk factors for the disease. We also stratify HC individuals into risk groups on the basis of CSF biomarkers for the two hallmark AD pathologies. Results show that the broad category of MCI can be decomposed into subsets of individuals with significantly different average regional atrophy rates. By thus selectively identifying individuals, combinations of these biomarkers and risk factors could enable significant reductions in sample size requirements for clinical trials of investigational AD-modifying therapies, and provide stratification mechanisms to more finely assess response to therapy. Power is sufficiently high that detecting efficacy in MCI cohorts should not be a limiting factor in AD therapeutics research. In contrast, we show that sample size estimates for clinical trials aimed at the preclinical stage of the disorder (HCs with evidence of AD pathology) are prohibitively large. Longer natural history studies are needed to inform design of trials aimed at the presymptomatic stage.

## Introduction

There is increased interest in Alzheimer disease (AD) clinical trials focusing on the predementia stages of the disease, particularly the preclinical stage [1]–[4]. This has been spurred by the growing understanding that AD follows an insidious course with pathologies developing over periods of decades prior to dementia onset [5]–[7]; by the establishment of biomarkers that can show the presence of AD pathologies in the early phases of the disorder [8]–[11]; and by the failure of inhibiting [12]–[15] and clearing [16] agents for one of the pathologies, amyloid, to produce cognitive improvement in trials involving participants with mild clinical AD. The etiology of AD, however, remains unknown and the defining pathologies of the disease occur also in other disorders and to varying degrees in the course of normal aging [5], [17]–[20]. This has lead to difficulty in confidently identifying individuals who are in the earliest stages of the disorder. Moreover, disease-related rates of change for clinical, cellular, and structural measures are significantly lower in the predementia stages. Yet predementia clinical trials require appropriately selected participants – especially given potentially serious side effects of many therapies – and outcome measures that will be sensitive to the subtle changes that occur in the earliest stages of the disease. The development of predementia trials has been hampered by the compounding difficulties in satisfying these two issues. Preventive trials in particular, involving cognitively intact participants [21], pose a considerable challenge because of increased uncertainty that the participants are on an AD trajectory, and because disease-related rates of change are very low in the presymptomatic stage, potentially necessitating trials of much longer duration than have hitherto been performed.

There are three main pathologies associated with AD: tau pathology, amyloid pathology, and neuronal injury [22]. The primary lesions associated with tau pathology are intraneuronal neurofibrillary tangles (NFTs), composed of phosphorylated tau proteins (ptau). The primary lesions associated with amyloid pathology are extraneuronal aggregates of fibril amyloid-beta_1_42_ (Aβ) proteins, which become neuritic and often contain ptau [23]. Tau pathology, however, has been found to be universally present in normal aging [5], and amyloid pathology, though not universal, is highly prevalent in the elderly [24]. Additionally, these pathologies are found at elevated levels in many brain diseases [20]. Nevertheless, though not specific to AD, the density and distribution of NFTs and Aβ plaques are the defining features of AD neuropathologic changes [25], with associated neuronal dysfunction and loss producing clinical decline and dementia.

Neuropathological and biomarker studies have demonstrated that both tau and amyloid pathologies develop over a long time frame prior to onset of clinical symptoms. In the preclinical phase of AD, NFTs initially appear in the transentorhinal region, then spread through limbic cortex, before spreading to association cortex, then to primary motor and sensory cortices as the disease progresses to the most severe stage [6]. Amyloid deposits appear initially in the basal portions of the frontal, temporal and occipital lobes but become widespread across the cortex [6]. Thus, tau and amyloid pathologies are known to show distinct temporal and topographic patterns of development in the early stages of the disease, and ultimately are widespread throughout the cortical mantle. The sequence in which elevated tau and amyloid pathologies become indicative of incipient AD, however, is the subject of current debate [26]–[28].

Biomarkers of brain tau and amyloid pathology can be obtained from cerebrospinal fluid (CSF) [9], [10]. As amyloid becomes sequestered into plaques in the brain, the concentration of Aβ proteins in CSF decreases. As tau pathology increases in the brain, the concentration of tau and ptau proteins increases in CSF. In patients with mild cognitive impairment (MCI) [29] the presence of these biomarkers is associated with a higher risk of developing dementia [30]–[33].

Baseline atrophy as detected on structural MRIs is also known to predict AD development [34]–[38]. Structural MRI is sensitive to brain changes that occur in normal aging [39]–[42], with rates of change accelerating as cognitive symptoms develop and worsen [43]. Although structures in the medial temporal lobe, including the entorhinal cortex, hippocampus, and amygdala, are most affected by AD, atrophy is widespread across the cortex, even in the prodromal phase [44]. Several research groups have shown that patterns of regional atrophy across the cortex can reliably differentiate patients with mild AD from healthy older controls, and that the degree of atrophy in these regions is predictive of the development of dementia in patients with MCI [45]–[48]. We have previously shown that relative to sample size requirements for clinical trials that used current MCI criteria, constraining enrollment to MCI participants showing a pattern of regional atrophy characteristic of mild AD would enable substantial sample sizes reductions [49].

In addition to affording enrichment strategies by improving identification of individuals at high risk of decline, measures of brain atrophy on structural MRIs can also prove useful as outcome measures. The standard clinical outcome measures for AD clinical trials have been designed for use in trials with dementia patients and are relatively insensitive to changes that occur in the predementia stage. Additionally, clinical measures may be influenced by symptomatic changes as well as by disease-modifying effects of therapy. Atrophy rates from serial MRI, which are sensitive to changes that occur in the predementia phase and which show lower inter-individual variability than clinical measures [50], [51], can be used as outcome measures to increase trial power [49], [52], while providing an evidentiary setting to support disease-modifying claims for therapy.

CSF and structural MRI biomarkers provide complementary information [38], [53]–[56], and when used together, improve prediction of dementia in individuals with MCI [56], [57]. Although some studies have shown the potential value of enriching clinical trials in the MCI phase based on biomarker status [58], [59], none have systematically compared the relative value of clinical measures, CSF biomarkers, and disease-specific atrophy biomarkers individually and together.

Individuals with MCI retain relatively high cognitive function, and slowing or arresting the disease in this population offers immense benefits [60]. To explore sample size requirements for clinical trials aimed at this population, we examined enrichment strategies based on CSF and MRI biomarkers to identify MCI individuals who are most likely to experience decline over the course of a clinical trial, and examined the relative ability of subregional and whole brain volume MRI outcome measures to enable further sample size reductions. To assess the relative powers for outcome measures and enrichment choices, we performed statistical significance testing for multiple pair-wise comparisons of outcome measures for different enrichment strategies, and for multiple pair-wise comparisons of enrichment strategies for different outcome measures.

There is, however, growing concern that by the time individuals experience noticeable cognitive impairment and brain atrophy, therapies may be too late to stop the neurodegenerative cascade [3]. Thus, preventive trials focused on asymptomatic individuals with biomarker evidence of AD pathology – and who therefore may be in a preclinical phase of the disorder – are being considered. To determine the feasibility of such trials, we also assessed rate of clinical decline and regional brain atrophy in cognitively healthy (HC) individuals who are likely to be in a presymptomatic stage of AD, based on CSF biomarkers. We considered HCs with CSF evidence of both amyloid and tau pathology as those most likely at risk for developing AD since prior studies have shown that CSF Aβ is associated with elevated entorhinal cortex atrophy rate and elevated clinical decline only in the presence of elevated CSF ptau [61], [62]. We calculated sample sizes based on the observed rates of change in the HC group that tested positive for both measures, relative to the control group of stable HCs who tested negative for CSF Aβ.

## Methods

We examined participants from the Alzheimer’s Disease Neuroimaging Initiative (ADNI, www.adni-info.org). Relevant details of ADNI, including participant enrollment criteria, MR image acquisition, and CSF collection and analysis methods are provided in File S1.

### Participants

We evaluated 390 older participants, divided into two predementia groups and a control group. Since ultimately both amyloid and tau pathologies are necessary concomitants for AD diagnosis, HCs most likely to be in a preclinical stage of AD are those who show CSF evidence for both amyloid and tau pathologies (Aβ^+^Ptau^+^ HCs; see below for definition for positive Aβ and ptau status). Thus, one predementia group comprised the 21 Aβ^+^Ptau^+^ HCs; one of these HCs progressed to AD by 36-months, while two others progressed to MCI by 24-months. The other predementia group comprised 311 MCI participants. The control group comprised 58 HC participants with longitudinally stable HC diagnosis and CSF biomarker evidence suggesting no amyloid pathology (Aβ^–^ HCs). We also examined atrophy rates in Aβ^+^ HCs with respect to the control group, and compared with the results of others that examined similar dichotomization. HC participants, Table 1, were evaluated at 0, 6, 12, 24, and 36 months; MCI participants, Table 2, were additionally evaluated at 18 months. The research protocol was approved by each local institutional review board, and written informed consent was obtained from each participant. ADNI participant IDs are provided in File S2.

**Table 1:** Cognitively healthy participant demographic and baseline data.

HC Group	N	%/#Female	Age[years]	MMSE	ADAS-Cog	CDR-SB	Aβ[pg/ml]	Ptau[pg/ml]
HC Aβ^−^	58*	52/30	75.5 (5.3)	29.1 (1.1)	5.9 (2.8)	0.0 (0.1)	242.8 (25.9)	21.1 (8.0)
HC Aβ^+^	39	46/18	77.0 (5.3)	29.1 (1.0)	7.1 (3.1)	0.0 (0.1)	143.9 (27.7)	31.3 (18.4)
HC Aβ^+^Ptau^−^	18	50/9	75.1 (4.9)	29.0 (1.0)	6.9 (2.6)	0.0 (0.1)	146.8 (25.3)	17.1 (3.5)
HC Aβ^+^Ptau^+^	21	43/9	78.6 (5.1)	29.2 (0.9)	7.2 (3.6)	0.0 (0.0)	141.4 (30.1)	43.5 (17.2)

N is the number of participants; values with parentheses are mean (standard deviations). CDR-SB: Clinical Dementia Rating, sum of boxes score; ADAS-Cog: cognitive subscale of the Alzheimer’s Disease Assessment Scale; MMSE: Mini Mental State Exam. Aβ and ptau: cerebrospinal fluid (CSF) densities of these proteins (see also Table 3); HC: cognitively healthy; Aβ^+^ means ≤192 pg/ml; ptau^+^ means ≥23 pg/ml.

* Excluding two who had converted to MCI at 24-months.

**Table 2:** MCI participant demographic and baseline data.

MCI group	N	%/#Female	Age[years]	MMSE	ADAS-Cog	CDR-SB	Aβ[pg/ml]	Ptau[pg/ml]
All	311‡	37/117	74.8 (7.4)	27.0 (1.8)	11.6 (4.3)	1.6 (0.9)	162.2 (52.9)*	35.8 (17.2)$
Aβ^−^	39*	31/12	75.3 (9.1)	27.2 (1.9)	10.7 (4.7)	1.3 (0.7)	245.9 (26.0)	20.7 (8.5)
Aβ^+^	127*	36/46	74.3 (6.8)	26.8 (1.8)	12.2 (4.5)	1.6 (0.9)	136.5 (25.0)	40.4 (16.6)
Ptau^−^	47$	26/12	75.7 (7.7)	27.2 (1.8)	10.0 (4.4)	1.5 (0.9)	211.3 (56.2)	17.4 (3.4)
Ptau^+^	120$	38/46	74.2 (7.3)	26.8 (1.8)	12.6 (4.5)	1.6 (0.9)	142.8 (36.3)$	43.0 (15.0)
MRI^−^	153‡	34/52	74.8 (7.5)	27.3 (1.7)	9.9 (3.7)	1.4 (0.7)	176.1 (60.0)∧	30.8 (16.3)∧
MRI^+^	156‡	41/64	74.8 (7.3)	26.7 (1.7)	13.2 (4.3)	1.7 (1.0)	151.2 (44.0)∧	40.1 (17.0)∧
Aβ^−^Ptau^−^	31	26/8	75.1 (8.6)	27.3 (1.8)	9.7 (4.1)	1.3 (0.7)	245.9 (27.5)	17.3 (3.7)
Aβ^+^Ptau^−^	16	25/4	75.2 (5.5)	27.1 (1.7)	10.5 (5.2)	1.8 (1.1)	144.3 (30.1)	17.8 (2.7)
Aβ^+^Ptau^+^	111	38/42	74.2 (7.0)	26.8 (1.8)	12.4 (4.4)	1.6 (0.9)	135.4 (24.1)	43.6 (15.2)
Aβ^+^MRI^−^	48	31/15	75.2 (7.0)	27.1 (1.8)	10.0 (3.8)	1.4 (0.6)	136.5 (27.0)	37.1 (17.0)
Aβ^+^MRI^+^	77	39/30	73.8 (6.7)	26.6 (1.7)	13.5 (4.4)	1.8 (1.1)	136.7 (24.2)	42.6 (16.3)
Aβ^−^Ptau^−^MRI^−^	22	32/7	73.5 (9.4)	27.5 (1.8)	8.6 (3.7)	1.3 (0.6)	245.4 (29.4)	17.6 (3.7)
Aβ^+^Ptau^−^MRI^−^	9	11/1	75.7 (5.0)	27.3 (1.4)	8.9 (3.3)	1.2 (0.7)	152.6 (27.1)	17.8 (2.3)
Aβ^−^Ptau^+^MRI^−^	5	40/2	76.8 (10.0)	27.6 (1.5)	12.2 (0.7)	1.1 (0.7)	251.8 (20.2)	28.2 (3.3)
Aβ^+^Ptau^+^MRI^−^	39	36/14	75.0 (7.4)	27.1 (2.0)	10.2 (3.9)	1.4 (0.5)	132.7 (25.9)	41.6 (15.6)
Aβ^+^Ptau^+^MRI^+^	71	38/27	73.8 (6.7)	26.7 (1.7)	13.7 (4.2)	1.7 (1.1)	136.9 (23.3)	44.7 (15.0)

See Tables 1 and 3 for key. MCI: mild cognitive impairment.

‡ Two MCI participants were not classified for MRI^+/−^ due to technical issues; both had cognitive data and were Aβ^+^; 1 was ptau^−^, the other ptau^+^.

* 166 (53.4%) of the 311 MCI subjects had CSF Aβ data. All have ptau; includes the two in ^‡^.

$ 167 (53.7%) of the 311 MCI subjects had CSF ptau data. One of these does not have Aβ data, but has cognitive data and is ptau^+^MRI^+^. The 167 include the two in ^‡^.

∧Only 75 MRI^−^ had CSF Aβ and ptau data; only 89 MRI^+^ had CSF Aβ data; 90 MRI^+^ had ptau data.

### CSF Measures

CSF data were available on approximately half the ADNI participants. We used previously established threshold concentrations of CSF Aβ and ptau to stratify MCI participants into risk groups: positive risk was defined as Aβ concentrations less than or equal to 192 pg/ml (Aβ^+^), and ptau concentrations greater than or equal to 23 pg/ml (Ptau^+^) [63].

### Clinical Measures

The Clinical Dementia Rating Scale, sum of boxes score (CDR-SB), a commonly used outcome measure in AD clinical trials was used to assess disease severity [64]–[67]. We examined change over time, relative to baseline, in this measure as a function of risk group.

### MRI Measures

We downloaded all available raw MRI data for each participant from the public ADNI website (loni.ucla.edu/ADNI/Data) and preprocessed all scans using image correction procedures for site-specific distortion effects updated for recent scanner changes [52]. We *qu*antified *a*natomical *r*egional *c*hange in serial MRIs using Quarc [50], [68], a recently developed method from our laboratory. We analyzed data from all available time points that passed local quality control; from all the ADNI participants with longitudinal MRIs, 10% of HC (21) and 16% of MCI (60) failed quality control, due primarily to motion artifacts, change in scanner model, or change in RF coil, as described in [52]. To enable a more consistent comparison between the clinical and structural MRI outcome measures, we restricted analysis to participant-visits for which both cognitive and MRI data were available (total = 1621 : 223 Aβ^–^ HC, 84 Aβ^+^Ptau^+^ HC, 1314 MCI). MCI participants had on average 3.2 (standard deviation 1.3) follow-up visits (min 1, max 5); Aβ^–^ HC participants had on average 2.8 (1.0) follow-up visits (min 1, max 4); and Aβ^+^Ptau^+^ HC participants had on average 3.0 (0.9) follow-up visits (min 1, max 4).

We investigated atrophy rates in several regions of interest (ROIs), and in whole brain volume since this is currently used as a secondary outcome measure in AD clinical trials. We examined the ROIs that are affected by neurofibrillary pathology early in the disease process [25]: the hippocampus (a proposed diagnostic biomarker [69] that has also been investigated as an outcome measure in clinical trials [70]), entorhinal cortex, parahippocampus, fusiform gyrus, amygdala, and the retrosplenial cortex (the isthmus portion of the cingulate gyrus). We also examined the middle temporal gyrus and the inferior parietal cortex, sites of early amyloid deposition.

We used baseline MRI measures to stratify MCI participants into high and low risk groups, as previously described in detail [37]. Briefly, in prior work, we performed a discriminant analysis using cortical and subcortical ROIs to differentiate ADNI’s HC from AD participants. We then applied the resulting model, which incorporated measures of atrophy from medial and lateral temporal areas, retrosplenial cortex, and orbitofrontal areas) to MCI participants, classifying them into those whose atrophy in these regions more strongly resembled that found in the AD group (positive risk, or MRI^+^) or that found in the HC group (negative risk, MRI^–^).

Methodological bias in image registration, leading to artifactually elevated effect sizes and reduced sample size estimates, remains a concern in the structural neuroimaging literature [50], especially given recent reporting [59], [71], [72] on earlier methodology and results known to be strongly biased [50], [73], [74], and recent reports [69], [75], [76] citing follow-up methodology and results that are ostensibly corrected for bias [74], [77] but in fact, as shown in [50], remain significantly biased. Several robust approaches to reducing or eliminating bias have been developed [78], [79]. Our explicitly inverse-consistent approach [68] essentially eliminates potential bias by combining forward and reverse image registrations, and has been assessed vis-à-vis other approaches [50].

### Sample Size Estimates

Using all available time-points per participant, we investigated atrophy rates and rates of clinical decline using a linear mixed effects model [50]. We estimated the sample size required to detect 25% slowing in mean rate of decline for a hypothetical disease-modifying treatment versus placebo for a 24 month, two-arm, equal allocation trial, with a 6-months assessment interval, with the requirement that the trial have 80% power to detect the treatment effect using a 2-sided significance level of 0.05. The power calculations, modeling linear change over time for each participant, were based on the mean rate of decline for the patient cohorts relative to the rate of decline experienced by the control group of diagnostically stable Aβ^–^ HCs [50]. This represents maximal estimates for the disease (or treatable) effect, since therapies aimed at AD are unlikely to affect rate of change experienced by healthy older individuals. We assessed estimated sample sizes per risk group using rates of change in CDR-SB and in various brain measures as outcome variables.

### Statistical Comparisons and Confidence Intervals

The significance of the differences in atrophy rates experienced by different pairs of risk groups were calculated using Satterthwaite’s method [80]. Calculation of the 95% confidence intervals (CIs) for sample size estimates was based on the joint a posteriori probability density function of the mixed effects model parameters, as we previously described in detail [50]. Two-sided significance (p-values) for pair-wise comparison of sample sizes resulting from different enrichment strategies for various outcome measures were calculated using the probability distribution for the difference between the sample sizes, as described in [50].

## Results

### Annual Rates of Decline

**Figure 1:**
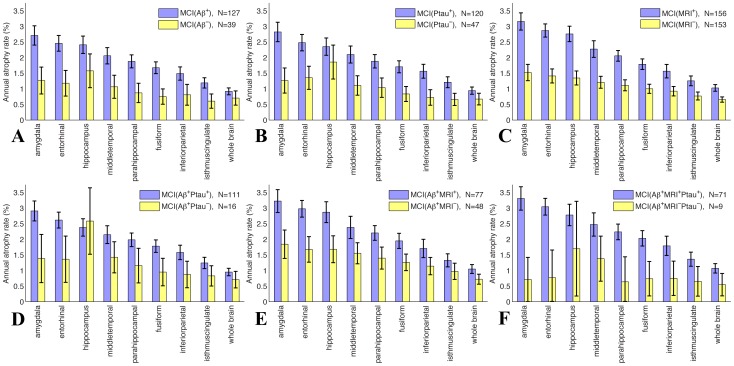
Annual atrophy rates for MCI participants, with 95% confidence intervals, for AD-relevant cortical and subcortical ROIs, grouped with respect to baseline Aβ, ptau, and volumetric MRI status (top row). In the bottom row, all participants are Aβ-positive. N is the number of participants. Numerical values are in File S1, Tables S1A–F.

Annual atrophy rates for MCI participants stratified into risk groups based on CSF and MRI biomarkers are shown for several cortical and subcortical ROIs in Figure 1. The upper row (A–C) shows differences in atrophy rates as a function of baseline biomarker status individually for CSF Aβ, CSF ptau, and regional atrophy. For each biomarker the high risk group showed substantially higher annual atrophy rates than the corresponding low risk group. Group differentiation was larger for subregional MRI measures, such as the amygdala, entorhinal, and hippocampus, than for whole brain volume (numerical values, with 95% CIs and p-values for the comparison between high and low risk groups for each ROI, are shown in File S1, Tables S1A–C). Baseline clinical scores, along with CSF and demographic data, for each risk group defined by individual biomarker status are shown in Table 2.

The lower row of Figure 1 (D–F) shows results of stratifying Aβ^+^ MCI participants into risk groups on the basis of measures of neuronal injury (ptau and atrophy). Aβ^+^ MCI participants who tested positive for either injury biomarker atrophied at a faster rate than those who tested negative (Figure 1D and E); even greater group differentiation was obtained when Aβ^+^ MCI participants were stratified on the joint presence of ptau and MRI injury biomarkers, though the number of individuals testing negative for both was very small (n = 9, Figure 1F). Numerical values, with 95% CIs and p-values for the comparison between high and low risk groups for each ROI, are shown in File S1, Tables S1D–F.

Annual atrophy rates were significantly higher for those Aβ^+^ MCI participants who tested positive for ptau as compared with those who tested negative for ptau for all subregions examined, except the hippocampus (Figure 1D). Although caution is needed in interpreting this unexpected result, due to the low number of Aβ^+^ MCI participants testing negative for ptau, we explored this further in post-hoc analyses. In File S1, Figure S1 and Table S3, we contrast annual atrophy rates for the 16 Aβ^+^Ptau^–^ MCI participants with those for the 31 Aβ^–^Ptau^–^ MCI participants. In Aβ^–^Ptau^–^ individuals, atrophy rates are relatively small and fairly uniform across ROIs; in contrast, Aβ^+^Ptau^–^ individuals show elevated atrophy rate for the hippocampus, with the difference between Aβ^–^Ptau^–^ and Aβ^+^Ptau^–^ individuals approaching significance (p = 0.075). For Aβ^+^Ptau^+^ individuals, however, all brain measures show significantly elevated atrophy rates compared with Aβ^–^Ptau^–^ individuals (File S1, Table S4).

**Figure 2:**
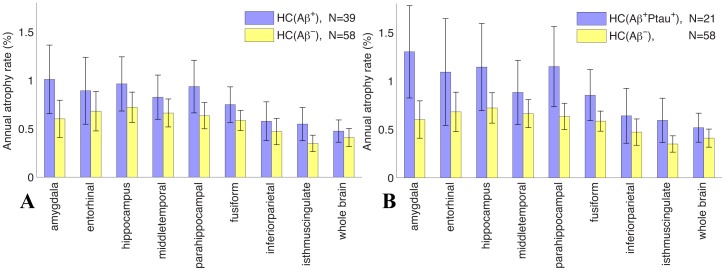
Annual atrophy rates for (A) Aβ^+^ HC participants, 4 of whom converted to MCI, and (B) Aβ^+^Ptau^+^ HCs participants (i.e., the HCs most likely to be preclinical-AD), 3 of whom converted to MCI, compared with the control group of stable Aβ^–^ HCs. N is the number of participants. Numerical values, including p-values, are in File S1, Tables S2A and S2B.

Figure 2B shows annual atrophy rates, for the same ROIs as in Figure 1, for the HCs with evidence of AD pathology (Aβ^+^Ptau^+^ HCs) and for the control group (note that the vertical scale in this figure is half that in Figure 1, reflecting the greater atrophy rates observed in MCI participants than in HCs). Although all ROIs show a clear trend for higher atrophy rates in Aβ^+^Ptau^+^ group as compared with controls, differences were small and significant only for the amygdala and parahippocampal gyrus, with the isthmus cingulate approaching significance (numerical values, with 95% CIs and p-values for the comparison between groups for each ROI, are shown in File S1, Tables S2B). The difference in annual rate of decline for CDR-SB between the Aβ^+^Ptau^+^ HCs and the controls approached significance: 0.25, CI = [0.04 to 0.45], vs. 0.04, CI = [0.01 to 0.07], p = 0.061. Figure 2A shows a comparison of atrophy rates for Aβ^+^ HCs with the control group. Differences approached significance for the amygdala and the parahippocampal gyrus, and reached significance for the isthmus cingulate (File S1, Table S2A).

### Sample Size Estimates

**Figure 3:**
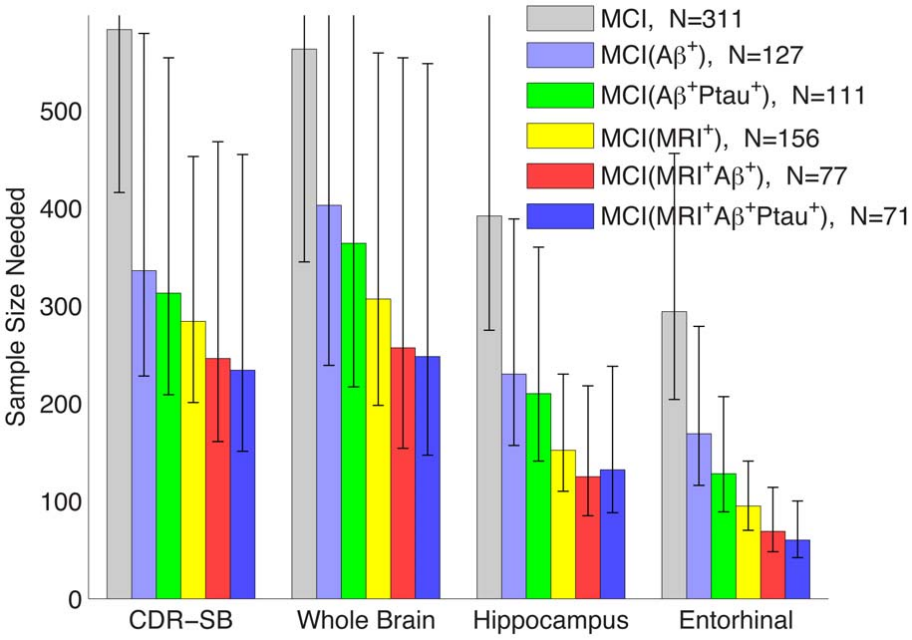
Estimated sample sizes, per arm, to detect a 25% reduction in annual rate of change in MCI participants under several enrichment strategies, relative to the annual rate of change in amyloid-negative stable HCs, at the p<0.05 level with 80% power assuming a 24 month trial with scans every six months. Sample sizes are estimated using a linear mixed effects model with fixed intercepts (no relative change at baseline) and random slopes applied to all data available up through 36 months. Error bars show the 95% confidence intervals. N is the number of participants. All numerical values are shown Table 3; p-values for comparisons are in Tables 4 and 5.

Figure 3 and Table 3 show sample size estimates with 95% CI for clinical trials enrolling MCI participants using the ADNI MCI criteria (all MCI) or for enriched trials targeted at MCI patients who test positive for one or more disease biomarker. Table 4 shows the two-sided significance (p-values) of the sample size reduction afforded when comparing pairs of enrichment strategies for various outcome measures (CDR-SB, whole brain atrophy, or regional atrophy). For the full MCI cohort (“All” column in Table 3), estimated sample size, per arm, to detect 25% slowing in rate of decline on CDR-SB was n = 583, with 95% CI = [416 to 894]. Restricting enrollment to MCI participants testing positive for Aβ and ptau would enable a 46% reduction in sample size for this measure compared with the full MCI cohort (n = 313 [209 to 554], p = 0.064). A larger, 51%, reduction in sample size would be afforded by selectively enrolling MCI patients with the AD regional atrophy pattern at baseline (MRI^+^), regardless of other risk factors (n = 284 [201 to 453], p = 0.015). Constraining enrollment to those testing positive for atrophy (MRI^+^) and amyloid (Aβ^+^) would enable even greater sample size reduction, 58% (n = 246 [161 to 468], p = 0.021).

**Table 3:** MCI sample size estimates for structural measures and a clinical measure – without enrichment, and with respect to five enrichment strategies.

Measures	All	Aβ^+^	Aβ^+^Ptau^+^	MRI^+^	MRI^+^Aβ^+^	MRI^+^Aβ^+^Ptau^+^
Entorhinal	294 [204 456]	169 [116 279]	128 [89 207]	95 [70 141]	69 [48 114]	60 [42 100]
Amygdala	306 [221 460]	184 [128 300]	144 [101 233]	122 [90 181]	102 [71 174]	93 [64 159]
Hipp	392 [275 608]	230 [157 389]	210 [141 360]	152 [110 230]	125 [85 218]	132 [88 238]
Parahipp	403 [276 654]	238 [159 410]	195 [132 333]	149 [107 228]	118 [80 204]	117 [79 209]
Fusiform	448 [307 723]	252 [168 432]	203 [137 346]	202 [143 320]	159 [105 291]	136 [90 249]
Isthmus cing	497 [339 810]	324 [212 586]	302 [195 560]	298 [202 504]	235 [148 470]	230 [142 472]
Whole Brain	563 [345 1091]	403 [239 851]	364 [217 773]	307 [198 559]	257 [154 554]	248 [147 548]
CDR-SB	583 [416 894]	336 [228 579]	313 [209 554]	284 [201 453]	246 [161 468]	234 [151 455]

The 95% confidence intervals of the estimated sample sizes are shown in brackets. Sample size estimates are those required to detect 25% slowing in the rate of change in MCI (under various enrichment strategies) that is in excess of that seen in Aβ-negative (Aβ^−^) HCs (N = 58). P-values for selected pair-wise comparisons are in Tables 4 and 5.

Key: All = all MCI participants (no enrichment); Aβ^+^ = MCI participants who are Aβ-positive at baseline; Aβ^+^Ptau^+^ = MCI participants who are both Aβ- and Ptau-positive at baseline; MRI^+^ = MCI participants who have AD-like atrophy at baseline; MRI^+^Aβ^+^ = MCI participants who are Aβ-positive and have AD-like atrophy at baseline; MRI^+^Aβ^+^Ptau^+^ = MCI participants who are simultaneously positive for all three biomarkers; Hipp = Hippocampus; Parahipp = Parahippocampus; cing = cingulate; CDR = clinical dementia rating – sum of boxes score. See text for definition of positive biomarker status classification.

**Table 4:** P-values for significance of difference in sample size estimates (Table 3, Figure 3) from pairs of enrichment specifications (rows) for particular measures (columns).

Enrichments	CDR	Brain	Hipp	Amyg	Erc
All vs. Aβ^+^	0.089	0.45	0.097	0.088	0.081
All vs. Aβ^+^Ptau^+^	0.064	0.33	0.057	**0.012**	**0.007**
All vs. MRI^+^	**0.015**	0.12	**7×10^−4^**	**5×10^−4^**	**3×10^−5^**
All vs. MRI^+^Aβ^+^	**0.021**	0.088	**9×10^−4^**	**8×10^−4^**	**6×10^−6^**
All vs. MRI^+^Aβ^+^Ptau^+^	**0.018**	0.081	**0.003**	**3×10^−4^**	**<10^−6^**
Aβ^+^ vs. Aβ^+^Ptau^+^	0.83	0.83	0.78	0.42	0.36
Aβ^+^ vs. MRI^+^	0.58	0.5	0.15	0.13	**0.037**
Aβ^+^ vs. MRI^+^Aβ^+^	0.41	0.33	0.074	0.071	**0.005**
Aβ^+^ vs. MRI^+^Aβ^+^Ptau^+^	0.34	0.31	0.12	**0.038**	**0.002**
Aβ^+^Ptau^+^ vs. MRI^+^	0.75	0.67	0.27	0.53	0.28
Aβ^+^Ptau^+^ vs. MRI^+^Aβ^+^	0.53	0.45	0.13	0.29	0.051
Aβ^+^Ptau^+^ vs. MRI^+^Aβ^+^Ptau^+^	0.45	0.42	0.19	0.18	**0.019**
MRI^+^ vs. MRI^+^Aβ^+^	0.7	0.69	0.55	0.58	0.29
MRI^+^ vs. MRI^+^Aβ^+^Ptau^+^	0.61	0.64	0.69	0.38	0.13
MRI^+^Aβ^+^ vs. MRI^+^Aβ^+^Ptau^+^	0.9	0.95	0.87	0.76	0.67

Values significant at the 5% level are underlined and bold. See Table 3 legend for key. Amyg = Amygdala; Erc = entorhinal cortex. Calculation of p-values described in [50].

Table 5 shows the significance (p-value) of the differences in sample size estimates when comparing different outcome measures. For an unenriched trial, adequate power to detect slowing in annual rate of atrophy in the entorhinal cortex would be obtained from a trial with n = 294 [204 to 456] MCI participants per arm, versus n = 583 [416 to 894] per arm to detect slowing in annual decline on CDR-SB (p = 0.015, Table 5). Stratifying by risk factor further reduces estimated sample sizes. For example, using atrophy rate in the entorhinal cortex as the outcome variable, a sample size as small as n = 128 [89 to 207] participants per arm was obtained for Aβ^+^Ptau^+^ participants, a significant reduction (78%, p = 0.007, Table 4) relative to the undifferentiated MCI cohort. Even greater reduction is provided by restricting enrollment to MRI^+^ participants (n = 95 [70 to 141], p = 3×10^−5^), or to MRI^+^Aβ^+^Ptau^+^ participants (n = 60 [42 to 100], p<10^−6^).

**Table 5:** P-values for significance of difference in sample size estimates (Table 3, Figure 3) from pairs of measures (rows) using particular enrichment specifications (columns).

Measures	All	Aβ^+^	Aβ^+^Ptau^+^	MRI^+^	MRI^+^Aβ^+^	MRI^+^Aβ^+^Ptau^+^
CDR vs. Brain	0.92	0.64	0.7	0.81	0.92	0.88
CDR vs. Hipp	0.16	0.25	0.24	**0.023**	0.056	0.12
CDR vs. Amyg	**0.017**	0.058	**0.016**	**0.002**	**0.011**	**0.009**
CDR vs. Erc	**0.015**	**0.033**	**0.005**	**4×10^−5^**	**2×10^−4^**	**8×10^−5^**
Brain vs. Hipp	0.28	0.14	0.15	**0.022**	0.062	0.11
Brain vs. Amyg	0.061	**0.032**	**0.01**	**0.002**	**0.015**	**0.01**
Brain vs. Erc	0.052	**0.019**	**0.004**	**7×10^−5^**	**3×10^−4^**	**1×10^−4^**
Hipp vs. Amyg	0.37	0.48	0.23	0.39	0.54	0.3
Hipp vs. Erc	0.31	0.33	0.12	0.067	0.064	**0.018**
Amyg vs. Erc	0.86	0.78	0.68	0.32	0.21	0.18

Values significant at the 5% level are underlined and bold. See Tables 3 and 4 legends for key.

Calculation of p-values described in [50].

For Aβ^+^Ptau^+^ HCs, sample size estimates for all ROIs and CDR-SB were prohibitively large. For example, using the amygdala as an outcome measure, we found an estimated sample size of n = 773 participants per arm, with 95% CI = [256 to 34673]; for the entorhinal cortex, the estimate was n = 2672 participants per arm, CI = [453 to >100000]; for the hippocampus, the estimate was n = 1763 participants per arm, CI = [400 to >100000]. For the CDR-SB, we found an estimated sample size of n = 1284 participants per arm, CI = [333 to >100000]. Since the extremely large upper bounds in the CIs renders these rate-of-change measures ineffective as outcome measures in longitudinal trials of standard duration we computed sample size estimates for a trial duration of five years, assuming constant annual rates of decline. As expected, this did not substantially alter these results.

## Discussion

Here we show that stratifying MCI participants into dichotomized categories with respect to established AD biomarkers results in subgroups of participants with different rates of clinical decline and brain atrophy, and correspondingly different potentially treatable effect sizes that can be leveraged to increase the efficiency of clinical trials. We further show that power for detecting change due to disease progression varies by outcome measure, so that the most powerful outcome measure-enrichment strategy combination dramatically enhances the ability to detect therapeutic effects of investigational disease-altering treatments. In contrast, when using CSF biomarkers to identify at-risk individuals in the asymptomatic stage, though small differences in atrophy rates relative to the control group were found for restricted brain regions, even reaching significance for the amygdala and parahippocampal cortex, the variance relative to the small effect size suggests that preventive trials using the most sensitive atrophy rate measure, let alone the standard clinical measure, would be prohibitively large, owing to the extremely high upper bounds on the sample size estimates.

As has long been known, the diagnosis of MCI does not reflect a homogenous etiology, but is composed of individuals who may suffer from cognitive impairment due to a variety of causes, including AD pathology. Even among those with AD pathology, individuals are at different stages along the disease continuum, with corresponding differences in rate of expected decline. Given this heterogeneity, clinical trials aimed at the prodromal phase can benefit greatly from enrichment strategies that selectively enroll individuals on the basis of biomarker evidence of disease pathology. Not only can this ensure that enrolled individuals show the pathology that is targeted by the therapeutic agent under investigation (though Aβ pathology is most commonly targeted [81], therapies aimed at tau are also under investigation [82], [83]), it can also aid in the identification of individuals at increased risk of rapid disease progression, thereby enabling smaller and shorter duration trials. Alternatively, without enrollment restriction, biomarker stratification could enable potentially informative subgroup analyses.

In addition to providing a basis for clinical trial enrichment, structural MRI measures of change have emerged as the most promising biomarkers for detecting effects of therapy – beneficial or adverse – in AD clinical trials [84]. They sensitively track the disease state, with rates of atrophy tending to accelerate as the disease progresses from preclinical to early AD dementia [43], [85], with regional rates of atrophy showing higher sensitivity than whole brain and clinical measures [50]. Here, we observed that of the subregional measures, atrophy rate of the entorhinal cortex consistently provided the smallest estimated sample size, regardless of enrichment strategy. Atrophy rate for the amygdala was the next most powerful outcome measure, although sample size estimates obtained using this measure did not significantly differ from those obtained using the entorhinal or the hippocampus as outcome measures. The relatively high power for rate of decline of the amygdala is in agreement with recent reports indicating that the amygdala is prominent in early AD [50], [86], [87]. However, caution is warranted in interpreting relative importance of the amygdala versus the hippocampus because of possible mislabeling of voxels for these ROIs due to their proximity and similar image contrast.

In contrast to MCI, there is a relatively high degree of similarity in rate-of-change outcome measures for HCs who may be in a preclinical stage of AD (those testing positive for CSF Aβ and ptau) and those unlikely to be in a preclinical stage of AD (those testing negative for CSF Aβ). Studies to date have not presented a clear picture on how amyloid is associated with increased brain atrophy rates in HCs. Bourgeat et al [88] found that hippocampal atrophy was associated with β-amyloid deposition in the inferior temporal neocortex, as measured by PiB retention in PET imaging. Chételat et al [89] recently found accelerated cortical atrophy, particularly in the middle temporal gyrus though not in medial temporal lobe structures, in cognitively normal elderly with PiB evidence of high β-amyloid deposition. It should be noted that cortical ‘atrophy’ averaged over the 54 PiB-negative participants appears to show large areas of the cortex *expanding*, particularly in sulcal regions (Figure 1 [89]), a biologically implausible effect that calls into question the accuracy of the method for serial MRI analysis; effects that rely on differences between a study cohort and a control cohort, as in [89], should not be affected by additive bias, but recent findings of bias in image registration point to the need for establishing fidelity of longitudinal image analysis methods [50], [74]. Earlier, Fjell et al [42] showed that in HCs with low levels of CSF Aβ, cortical atrophy rates were significantly correlated with CSF Aβ, particularly in regions not vulnerable in the early stages of AD. Desikan et al observed that atrophy rate in entorhinal cortex was associated with CSF Aβ only in the presence of ptau [62]. Dickerson et al [90] showed that a baseline MRI signature for AD – developed in a non-ADNI cohort – that was predictive of subsequent clinical decline in HCs was also associated with decreased CSF Aβ in HCs. Note that care must be taken when comparing results based on PiB, which binds to the neuritic – though not diffuse – amyloid plaques, and CSF Aβ for three reasons: (1) the CSF Aβ values are amyloid monomer concentrations [63], [91]–[93], whereas PiB values reflect density of plaques composed of amyloid fibrils; (2) CSF Aβ is a global, not a local or regional measure of amyloid; (3) they are not correlates, but rather have different distributions with age, as shown in [94], [95]. Nevertheless, in the current study, a significantly elevated atrophy rate for CSF Aβ^+^ HCs relative to CSF Aβ^–^ HCs was observed only in the isthmus cingulate (File S1 Table S2A). Atrophy rate in the parahippocampal gyrus and amygdala was significantly elevated in those additionally testing positive for ptau (File S1 Table S2B).

The small difference in atrophy rates and rates of clinical decline observed here between HCs testing positive for CSF biomarkers and those testing negative imply that clinical trials, even if of longer duration than the typical 18 to 24 months, will lack power to detect treatment effects using currently available clinical or structural outcome measures. This conclusion is seemingly at odds with the results of a recent study by Schott and colleagues [96] which reported that brain atrophy may be a useful outcome measure in preventive trials. In that study ADNI’s HCs were categorized with respect to CSF Aβ, using the same cut-off threshold applied here, and sample sizes estimated based on rate of atrophy of whole brain, hippocampus, and ventricles, using baseline and 12-month follow-up MRIs only; whole brain atrophy rate was calculated using the KN-BSI method [97], HMAPS with BSI [98] was used for the hippocampus, and BSI was used for the ventricles. Results showed that for a treatment effect reported to be equal to 48% of a disease effect calculated from rates of change in 40 Aβ^+^ HCs relative to rates of change in 65 Aβ^–^ HCs, sample size of 141 [86 to 287] participants per arm for whole brain atrophy as the outcome measure and 467 [197 to 2675] participants per arm for hippocampal atrophy as the outcome measure would provide 80% power at a significance of 0.05. However, few clinical trials are powered on the basis of such a large effect size; most studies estimate sample sizes to provide sufficient power to detect a slowing in the disease-related rate of decline of 20% [99] or 25% [97] as we have done here. Scaling Schott and colleagues’ results to an effect size of 25% slowing in disease-related atrophy, to enable comparison with this and prior studies, yields sample size estimates of 500 [317 to 1058] participants per arm for whole brain atrophy as an outcome, and 1722 [726 to 9861] participants per arm for hippocampal atrophy as an outcome. Though the large sample size, and large upper confidence interval, renders hippocampal atrophy rate unsuitable for use as an outcome measure in a preclinical treatment trial, this analysis suggests that whole brain atrophy could be a feasibly outcome measure in a large preclinical trial. However, there is another important difference in the analysis methods that must be considered. Schott and colleagues estimated sample sizes using two timepoints only: baseline and a single followup at 12 months. More reliable estimates of atrophy rates and associated variances, and sample sizes derived from these, would come from using all available followup timepoints – of which there are up to four covering up to 36 months per HC participant – as we have done here. When we analyzed publicly available quality-controlled KN-BSI data for all available visits, as described in detail in [50], for the 39 Aβ^+^ HCs (including 4 converters) and 65 Aβ^–^ HCs (excluding 2 converters) available, we obtained a sample size estimate for whole brain atrophy of 1179 [375 to 33090] per arm. We note that, as a check we also analyzed the publicly available KN-BSI data using the baseline and 12 month time points only, and obtained an estimated sample size of 663 [307 to 2358] for 30 Aβ^+^ HCs (including 2 converters) and 53 Aβ^–^ HCs (excluding 1 converter). This estimate is in reasonable agreement, given the smaller number of subjects available for our analysis, with the results of Schott and colleagues [96] after translation to an effect size of 25% slowing in disease related atrophy (sample size of 500 [317 to 1058] per arm). The sample size of 1179 [375 to 33090] participants per arm, with the large upper bound on the 95% confidence interval when all available time points are used, indicates that rate of whole brain atrophy is not feasible as an outcome measure for AD prevention studies if the effect size of interest is 25% slowing of disease-related atrophy.

There is little information currently available on whether and how AD biomarkers change during the presymptomatic phase of the disease. Natural history studies of long duration will likely be required to establish estimates of biomarker trajectories in the presymptomatic phase so that estimates of the time to significant disease-related change can be established to inform needed duration of preventive clinical trials. Change in biomarkers of amyloid burden, which is thought to rise rapidly and subsequently rise more gently or even plateau during the predementia stage [88], [100]–[104], might provide sufficient power in a clinical trial of reasonable duration, if the period during which these changes occur can be reliably identified. Given the known temporal-topographic amyloid plaque deposition pattern, detecting anti-amyloid therapeutic efficacy might further be enhanced by use of longitudinal subregional measures of amyloid deposition from PET imaging, requiring cross-modality registration of structural MRI with PET images.

While current structural measures do not provide feasible outcome measures for primary prevention trials, they can significantly reduce sample sizes compared with cognitive outcome measures in secondary prevention trials, aimed at the prodromal phase when mild impairment is evident. Using enrichment strategies to selectively enroll individuals at high risk of imminent decline can reduce sample sizes even further. However, a strict enrichment approach to clinical trial design means screening out many candidate participants. In ADNI, only about 23% of the MCI cohort would satisfy screening criteria if restricted to those testing positive for all biomarkers examined here, Aβ, Ptau, and atrophy; 77% would fail screening, making this a challenging selective enrollment strategy. The reduced costs enabled by the gain in power from selectively enrolling fewer participants would need to be balanced against the increased cost of screening out large numbers of individuals. Furthermore, given general difficulties in recruiting subjects in clinical trials [105]–[108], particularly when they may be associated with deleterious side effects, a selective enrollment criterion that eliminated the majority of potentially eligible candidates could make it very difficult to recruit a large enough sample. Lorenzi et al. [58] explicitly assessed the screen-out cost for different single biomarker enrichment strategies, using change in ADAS-Cog and CDR-SB as outcome measures. They examined thresholds needed to either maximize inclusion of MCI-to-AD converters, or to minimize exclusion of these converters, where conversion took place within two years from baseline. The focus on participants who are known to convert in a short period, however, selects for younger participants [109] and shifts standard thresholds more into the AD-range (e.g., the CSF Aβ threshold is shifted from 192 pg/ml to 165.8 pg/ml); the more pronounced AD phenotype selected leads to substantial reductions in sample sizes at the cost of a high rate of screen failures. Strategies that minimized exclusion of converters rather than maximizing their inclusion resulted in larger sample sizes, though still smaller than that of an unenriched trial, with a more acceptable rate of screen failures. This study did not examine enrichment that could be enabled by combinations of biomarkers, or examine structural outcome measures, as we have done here.

In addition to weighing the costs of screen failures against improved trial power, ethical concerns must also be explicitly addressed during the design of a clinical trial that plans to incorporate an enrichment strategy [110]. In such trials, individuals are likely to be informed of their biomarker status, and it is not yet clear what implications that may have for an individual’s future. Institutional review boards will have to be convinced that the risks associated with disclosure of risk status are adequately minimized before such trials can proceed. With the increasing move towards preventive trials, in which risk must be defined on the basis of biomarkers, much attention is currently focused towards development of methods for accurately conveying information regarding biomarker risk to potential participants, while minimizing negative effects of learning one’s risk status.

An alternative approach to enrichment strategies, which would ease recruitment and avoid the necessity of informing participants of their risk status, is to enroll a broader set of individuals, drawing a balance between selectively enrolling those at high risk while minimizing screen failures, then stratifying participants into biomarker-defined subgroups for analyses. This could determine whether a treatment that might not be effective in the full group showed promise in identifiable subgroups. Such subgroup analyses, and enrichment, could result in drug labeling requirements by regulatory agencies limiting prescription of a successful agent to those with the biomarkers used in the trial. However, given the current lack of any effective therapy for delaying the disease, and the enormous burden the coming epidemic will place on society, establishing efficacy even in a small subgroup would be a development of major importance, and one that could be followed by future trials on less select populations.

A different approach to stratification and enrichment for reducing sample sizes for MCI and AD treatment trials was recently proposed that increased effect sizes by reducing inter-individual variance through adjustment for several factors, including age, genetics, clinical measures of disease severity, baseline brain measures, and CSF biomarkers [111]. The authors reported a 10–30% reduction in sample sizes with adjustment for 11 predefined variables. However, some variables might be identified as ‘nuisance’ variables [112], while others might be of crucial importance, depending on therapeutic targeting mechanisms. Thus, for example, if a treatment effect were found for a heterogeneous cohort, it could arise from a strong effect in a particular subset and little or no relevance or effect in another subset of participants. Therefore, though some ‘nuisance’ variability could be controlled for, subgroup analysis would still be needed to identify patients that might benefit most from a treatment, and those for whom risks might exceed the benefits.

A popular model of the sequence of AD biomarkers of the AD pathological cascade [27] postulates that amyloid deposition (and CSF Aβ-positivity [9], [94]) is an early event followed by neurofibirllary pathology (and CSF ptau-positivity [10]) – though this remains contentions [26], [28]. Since NFT pathology is strongly linked with synaptic and neuronal injury and loss, next in the postulated sequence of biomarkers is brain atrophy observable on MRI. Consistent with this, we found that in Aβ^+^ MCI individuals, annual atrophy rates were significantly higher for those who tested positive for ptau as compared with those who tested negative for ptau for all subregions examined, except the hippocampus. Interestingly, the hippocampus showed a trend for elevated atrophy rate earlier in the disease process, when evidence of Aβ pathology was present, but in the absence of ptau pathology. Although the statistical power is limited due to the low number of Aβ^+^Ptau^–^ MCI participants, and bearing in mind that CSF measures are global and so do not fully inform on pathology within particular subregions, a possible interpretation of these findings is that elevation of the hippocampal atrophy rate is an early event occurring during the progression from the initial Aβ^–^Ptau^–^ stage to the Aβ^+^Ptau^–^ stage, with more widespread atrophy occurring at a later stage, when ptau pathology becomes evident. This interpretation is not obviously at variance with the neuropathological evidence, which shows that the entorhinal cortex and hippocampus are both affected by NFT lesions in pre-clinical Braak stage II, additionally with scattered neuritic plaques appearing in the CA1 region [113], while substantial neuron loss for both regions appears to begin in later Braak stages when clinical symptoms manifest: 35% in the entorhinal cortex and 46% in CA1 [114], [115]. It is possible, perhaps likely, that the Aβ^–^Ptau^–^ MCI participants do not have prodromal AD, but that their cognitive impairment (and subsequent dementia in the case of the seven who converted to a diagnosis of “AD” during follow-up) is due to some other condition, such as vascular dementia or hippocampal sclerosis.

It is also interesting to note that annual atrophy rates for the 48 MCI Aβ^+^MRI^–^ participants are relatively high, almost 2% per year for the entorhinal, amygdala, and hippocampus (Figure 1), even though these participants do not exhibit a baseline atrophy pattern indicative of AD. However, 39 of these 48 participants are also Ptau^+^, indicating that neuronal injury is likely taking place [62]. Thus, although these participants have not yet lost substantial amounts of cortical tissues in AD-vulnerable areas, they are experiencing a rapid rate of degeneration in these areas.

A limitation of this study is that the ADNI HCs are not representative of the general population (although the MCI and AD cohorts have been shown to be representative of patients who might be recruited for therapeutic trials [116]). Effect sizes, therefore, between cognitively normal elderly Aβ^+^Ptau^+^ and Aβ^–^ individuals in a more representative sample might be different to those found here. Also, our sample size estimates did not model for screening failures or patient attrition, which can significantly affect trial design.

### Conclusion

Due to the failure of clinical trials of candidate disease modifying therapies to slow disease progression in patients already diagnosed with early AD, there is growing interest in conducting secondary and tertiary prevention trials and treatment trials for AD [1], [2], targeting cognitively healthy individuals exhibiting biomarker evidence of the disease and those with mild cognitive impairment. In addition to arresting or slowing clinical decline, establishing disease-modifying properties of therapies will require demonstrating an effect on disease biomarkers. Structural MRI measures of change have emerged as the most promising biomarkers for detecting effects of therapy. The dominant component to structural atrophy is neuron loss, prior to which there will be synapse loss and reduction in neuropil complexity. In the preclinical stage of AD, cognition remains intact, reflecting the preservation of neurons, and structural atrophy on MRI is minimally different from that in older individuals who are not in the preclinical stage. In contrast, cellular biomarkers for AD, indicating advancing amyloid and tau pathologies, become manifest during this stage. Based on the observed atrophy rates in the HCs most likely to have preclinical AD, sample size estimates for preclinical trials are prohibitively large. Longer natural history studies of HCs likely to progress to AD are needed to inform on potential strategies for evaluating treatment effects in this group. It will also be important to take cohort age into account, as larger disease-related effects would be expected with younger cohorts [109].

In contrast to the preclinical stage, effect sizes are large enough in MCI cohorts to render clinical trials quite feasible at this disease stage. However, given the heterogeneity in etiology and in rates of change in outcome measures across individuals categorized as MCI, enrichment in this disease stage offers important benefits. MCI participants testing positive for the AD atrophy pattern at baseline (MRI^+^) are likely to be more advanced along the disease trajectory than those testing negative. As a result, stratification by this measure alone offers the single strongest enrichment. However, our results show that the presence of either CSF Aβ or ptau biomarker, regardless of atrophy status, is associated with increased rates of change. Thus, selective enrollment of individuals with the targeted pathology for either anti-amyloid or anti-tau compounds would offer the additional advantage of increasing trial power. For trials aimed at other putative disease targets, where selective enrollment based on amyloid or tau pathology may not be desired, analyses may be stratified by these biomarkers to enhance power for detecting effects in subgroups and to more finely monitor response to therapy by disease stage.

CDR-SB is the most sensitive clinical outcome measure used in clinical trials, and its power is strongly enhanced by enrichment. However, several subregional ROIs, particularly the entorhinal cortex, amygdala, and hippocampus, are significantly more powerful than CDR-SB or whole brain volume, the MRI measure currently used as a secondary outcome variable in clinical trials. The power of subregional MRI outcome measures is also enhanced by enrichment. MRI outcome measures have yet to be validated as surrogates for clinical outcome measures, a process that will require successful clinical trials, but they provide strong evidence for disease-modifying – and not just symptomatic – claims for therapies. The sensitivity of these measures, as demonstrated here, suggests that *detecting* efficacy of candidate therapies in MCI participants is unlikely to be a limiting factor in AD therapeutics research.

## Supporting Information

File S1. (DOCX)

File S2. (CSV)
