# Supplementary material for: Enrichment and Stratification for Predementia Alzheimer Disease Clinical Trials
Source: PLoS One. 2012 Oct 17;7(10):e47739. doi: 10.1371/journal.pone.0047739 (PMC3474753; doi:10.1371/journal.pone.0047739)
Supplement: File S1. — (DOCX) [file pone.0047739.s001.docx]

**Supporting Information S1**

**
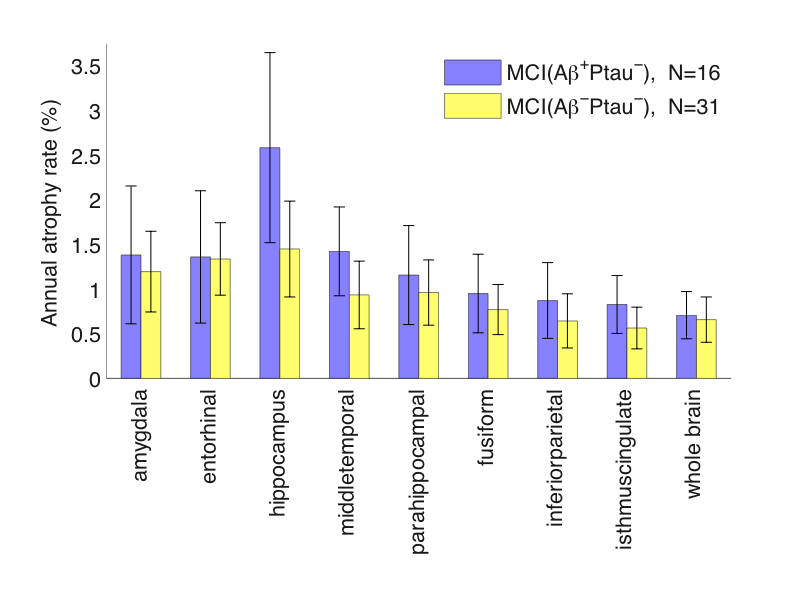
**

**Figure S1.** Annual atrophy rates for Aβ-positive ptau-positive versus Aβ-negative ptau-negative MCI participants, with 95% confidence intervals, for AD-relevant cortical and subcortical ROIs. N is the number of participants. Numerical values are in Table S3.

**
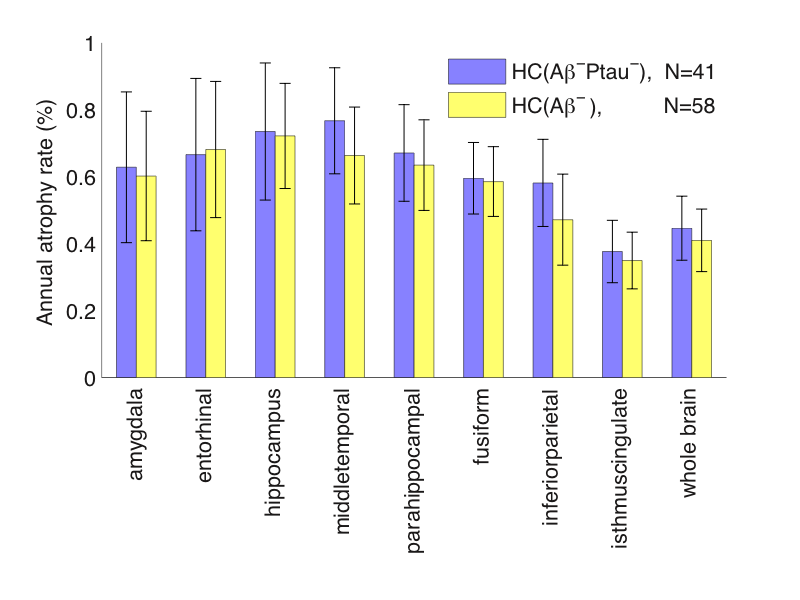
**

**Figure S2.** Two similar control groups, excluding 2 HC🡪MCI converters common to both.

**
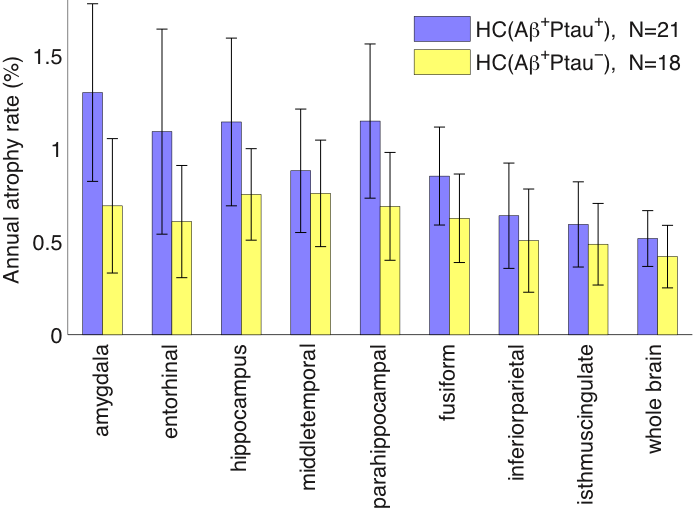
**

**Figure S3.** Atrophy rates in Aβ^+^ HCs with respect to ptau status (including 3 ptau^+^ and 1 ptau^−^ who converted to MCI). Numerical values are in Table S2C.

**
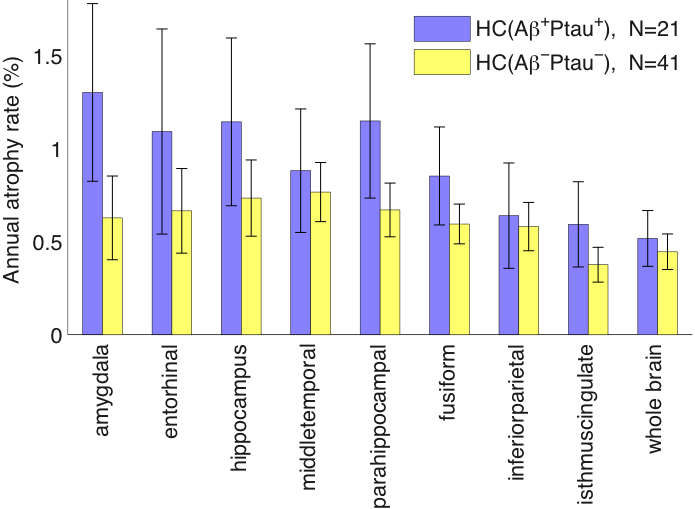
**

**Figure S4.** Atrophy rates in Aβ^+^ptau^+^ HCs (including 3 converters) compared with Aβ^−^ptau^−^ HCs (excluding 2 converters). Numerical values are in Table S2D.**Table S1A**

| Measure | Annual Atrophy  Rate (%)  MCI Aβ^+^  (N=127) | Annual Atrophy  Rate (%)  MCI Aβ^−^  (N=39) | p-val |
| --- | --- | --- | --- |
| Amygdala | 2.71 [2.40 3.02] | 1.26 [0.83 1.69] | **<10^-6^** |
| Entorhinal | 2.46 [2.20 2.71] | 1.18 [0.77 1.59] | **2x10^-6^** |
| Hippocampus | 2.41 [2.13 2.69] | 1.58 [1.03 2.12] | **0.009** |
| Middle temporal | 2.06 [1.80 2.32] | 1.06 [0.69 1.44] | **5x10^-5^** |
| Parahippocampal | 1.88 [1.67 2.09] | 0.87 [0.56 1.18] | **1x10^-6^** |
| Fusiform | 1.67 [1.49 1.86] | 0.75 [0.51 1.00] | **<10^-6^** |
| Inferior parietal | 1.49 [1.28 1.70] | 0.81 [0.47 1.14] | **0.001** |
| Isthmus cingulate | 1.19 [1.03 1.35] | 0.61 [0.38 0.83] | **1x10^-4^** |
| Whole brain | 0.92 [0.81 1.03] | 0.70 [0.48 0.93] | 0.1 |

p-values calculated using Satterthwaite’s method [[80](#_ENREF_80)].

**Table S1B**

| Measure | Annual Atrophy  Rate (%)  MCI Ptau^+^  (N=120) | Annual Atrophy  Rate (%)  MCI Ptau^−^  (N=47) | p-val |
| --- | --- | --- | --- |
| Amygdala | 2.82 [2.51 3.13] | 1.26 [0.86 1.67] | **<10^-6^** |
| Entorhinal | 2.48 [2.22 2.74] | 1.35 [0.98 1.72] | **5x10^-6^** |
| Hippocampus | 2.35 [2.07 2.63] | 1.86 [1.31 2.40] | 0.12 |
| Middle temporal | 2.10 [1.83 2.37] | 1.11 [0.80 1.42] | **7x10^-6^** |
| Parahippocampal | 1.88 [1.67 2.10] | 1.04 [0.73 1.35] | **3x10^-5^** |
| Fusiform | 1.70 [1.51 1.90] | 0.83 [0.59 1.08] | **<10^-6^** |
| Inferior parietal | 1.56 [1.34 1.78] | 0.72 [0.47 0.97] | **3x10^-6^** |
| Isthmus cingulate | 1.21 [1.04 1.38] | 0.66 [0.46 0.86] | **7x10^-5^** |
| Whole brain | 0.94 [0.83 1.06] | 0.67 [0.48 0.86] | **0.018** |

**Table S1C**

| Measure | Annual Atrophy  Rate (%)  MCI MRI^+^  (N=156) | Annual Atrophy  Rate (%)  MCI MRI^−^ (N=153) | p-val |
| --- | --- | --- | --- |
| Amygdala | 3.16 [2.88 3.43] | 1.52 [1.26 1.78] | **<10^-6^** |
| Entorhinal | 2.87 [2.66 3.08] | 1.41 [1.19 1.64] | **<10^-6^** |
| Hippocampus | 2.76 [2.51 3.01] | 1.35 [1.12 1.57] | **<10^-6^** |
| Middle temporal | 2.27 [2.01 2.54] | 1.21 [1.02 1.40] | **<10^-6^** |
| Parahippocampal | 2.06 [1.89 2.23] | 1.11 [0.94 1.29] | **<10^-6^** |
| Fusiform | 1.79 [1.62 1.96] | 1.00 [0.86 1.15] | **<10^-6^** |
| Inferior parietal | 1.56 [1.35 1.78] | 0.92 [0.77 1.07] | **3x10^-6^** |
| Isthmus cingulate | 1.26 [1.10 1.41] | 0.77 [0.64 0.90] | **4x10^-6^** |
| Whole brain | 1.03 [0.92 1.14] | 0.66 [0.57 0.74] | **<10^-6^** |

**Table S1D**

| Measure | Annual Atrophy  Rate (%)  MCI Aβ^+^Ptau^+^ (N=111) | Annual Atrophy  Rate (%)  MCI Aβ^+^Ptau^−^ (N=16) | p-val |
| --- | --- | --- | --- |
| Amygdala | 2.91 [2.59 3.23] | 1.38 [0.61 2.16] | **0.002** |
| Entorhinal | 2.62 [2.36 2.87] | 1.36 [0.62 2.10] | **0.006** |
| Hippocampus | 2.38 [2.10 2.66] | 2.58 [1.52 3.65] | 0.72 |
| Middle temporal | 2.15 [1.86 2.43] | 1.42 [0.93 1.92] | **0.02** |
| parahippocampal | 1.98 [1.76 2.20] | 1.16 [0.60 1.71] | **0.014** |
| Fusiform | 1.78 [1.58 1.98] | 0.95 [0.51 1.39] | **0.003** |
| Inferior parietal | 1.58 [1.35 1.81] | 0.87 [0.45 1.30] | **0.008** |
| Isthmus cingulate | 1.24 [1.06 1.42] | 0.83 [0.50 1.15] | **0.038** |
| Whole brain | 0.95 [0.83 1.07] | 0.71 [0.44 0.97] | 0.12 |

**Table S1E**

| Measure | Annual Atrophy  Rate (%)  MCI Aβ^+^MRI^+^ (N=77) | Annual Atrophy  Rate (%)  MCI Aβ^+^MRI^−^ (N=48) | p-val |
| --- | --- | --- | --- |
| Amygdala | 3.23 [2.86 3.59] | 1.84 [1.39 2.30] | **1x10^-5^** |
| Entorhinal | 2.98 [2.71 3.25] | 1.68 [1.27 2.08] | **1x10^-6^** |
| Hippocampus | 2.87 [2.53 3.20] | 1.68 [1.25 2.11] | **4x10^-5^** |
| Middle temporal | 2.38 [2.03 2.74] | 1.55 [1.21 1.89] | **0.001** |
| parahippocampal | 2.20 [1.97 2.44] | 1.40 [1.05 1.75] | **4x10^-4^** |
| Fusiform | 1.95 [1.71 2.19] | 1.26 [1.00 1.53] | **3x10^-4^** |
| Inferior parietal | 1.71 [1.41 2.00] | 1.14 [0.86 1.42] | **0.007** |
| Isthmus cingulate | 1.33 [1.12 1.54] | 0.97 [0.71 1.23] | **0.041** |
| Whole brain | 1.05 [0.91 1.19] | 0.72 [0.56 0.88] | **0.003** |

**Table S1F**

| Measure | Annual Atrophy  Rate (%)  MCI Aβ^+^MRI^+^Ptau^+^  (N=71) | Annual Atrophy  Rate (%)  MCI Aβ^+^MRI^−^Ptau^−^  (N=9) | p-val |
| --- | --- | --- | --- |
| Amygdala | 3.31 [2.93 3.68] | 0.71 [0.00 1.42] | **4x10^-5^** |
| entorhinal | 3.04 [2.78 3.31] | 0.77 [1.53 1.66] | **1x10^-3^** |
| hippocampus | 2.78 [2.43 3.12] | 1.70 [0.18 3.22] | 0.21 |
| Middle temporal | 2.47 [2.10 2.85] | 1.38 [0.66 2.10] | **0.021** |
| Parahippocampal | 2.24 [1.99 2.49] | 0.64 [1.26 1.44] | **0.005** |
| Fusiform | 2.03 [1.78 2.28] | 0.74 [0.19 1.29] | **0.001** |
| Inferior parietal | 1.79 [1.48 2.10] | 0.75 [0.20 1.30] | **0.007** |
| Isthmus cingulate | 1.36 [1.14 1.59] | 0.65 [0.18 1.13] | **0.023** |
| Whole brain | 1.07 [0.92 1.22] | 0.55 [0.19 0.90] | **0.023** |

**Table S2A**

| Measure | Annual Atrophy  Rate (%)  HC Aβ^+^  (N=39) | Annual Atrophy  Rate (%)  HC Aβ^−^  (N=58) | p-val |
| --- | --- | --- | --- |
| Amygdala | 1.01 [0.66 1.36] | 0.60 [0.41 0.80] | 0.052 |
| Entorhinal | 0.89 [0.55 1.24] | 0.68 [0.48 0.88] | 0.31 |
| Hippocampus | 0.96 [0.68 1.24] | 0.72 [0.56 0.88] | 0.15 |
| Middle temporal | 0.83 [0.60 1.06] | 0.66 [0.52 0.81] | 0.24 |
| Parahippocampal | 0.94 [0.66 1.21] | 0.63 [0.50 0.77] | 0.057 |
| Fusiform | 0.75 [0.57 0.93] | 0.59 [0.48 0.69] | 0.13 |
| Inferior parietal | 0.58 [0.38 0.78] | 0.47 [0.34 0.61] | 0.39 |
| Isthmus cingulate | 0.55 [0.38 0.72] | 0.35 [0.26 0.43] | **0.047** |
| Whole brain | 0.48 [0.36 0.59] | 0.41 [0.32 0.50] | 0.39 |

**Table S2B**

| Measure | Annual Atrophy  Rate (%)  HC Aβ^+^Ptau^+^  (N=21) | Annual Atrophy  Rate (%)  HC Aβ^−^  (N=58) | p-val |
| --- | --- | --- | --- |
| Amygdala | 1.30 [0.82 1.78] | 0.60 [0.41 0.80] | **0.013** |
| Entorhinal | 1.09 [0.54 1.64] | 0.68 [0.48 0.88] | 0.18 |
| Hippocampus | 1.14 [0.69 1.60] | 0.72 [0.56 0.88] | 0.095 |
| Middlet emporal | 0.88 [0.55 1.21] | 0.66 [0.52 0.81] | 0.25 |
| Parahippocampal | 1.15 [0.73 1.56] | 0.63 [0.50 0.77] | **0.03** |
| Fusiform | 0.85 [0.59 1.12] | 0.59 [0.48 0.69] | 0.075 |
| Inferior parietal | 0.64 [0.36 0.92] | 0.47 [0.34 0.61] | 0.3 |
| Isthmus cingulate | 0.59 [0.36 0.82] | 0.35 [0.26 0.43] | 0.061 |
| Whole brain | 0.52 [0.37 0.67] | 0.41 [0.32 0.50] | 0.24 |

**Table S2C**

| Measure | Annual Atrophy  Rate (%)  HC Aβ^+^Ptau^+^ (N=21) | Annual Atrophy  Rate (%)  HC Aβ^+^Ptau^−^ (N=18) | p-val |
| --- | --- | --- | --- |
| Amygdala | 1.30 [0.82 1.78] | 0.69 [0.33 1.05] | 0.054 |
| Entorhinal | 1.09 [0.54 1.64] | 0.61 [0.31 0.91] | 0.14 |
| Hippocampus | 1.14 [0.69 1.60] | 0.75 [0.51 1.00] | 0.15 |
| Middle temporal | 0.88 [0.55 1.21] | 0.76 [0.47 1.05] | 0.59 |
| Parahippocampal | 1.15 [0.73 1.56] | 0.69 [0.40 0.98] | 0.085 |
| Fusiform | 0.85 [0.59 1.12] | 0.63 [0.39 0.86] | 0.22 |
| Inferior parietal | 0.64 [0.36 0.92] | 0.51 [0.23 0.78] | 0.51 |
| Isthmus cingulate | 0.59 [0.36 0.82] | 0.49 [0.27 0.71] | 0.51 |
| Whole brain | 0.52 [0.37 0.67] | 0.42 [0.25 0.59] | 0.41 |

**Table S2D**

| Measure | Annual Atrophy  Rate (%)  HC Aβ^+^Ptau^+^ (N=21) | Annual Atrophy  Rate (%)  HC Aβ^−^Ptau^−^ (N=41) | p-val |
| --- | --- | --- | --- |
| Amygdala | 1.30 [0.82 1.78] | 0.63 [0.40 0.85] | **0.018** |
| Entorhinal | 1.09 [0.54 1.64] | 0.67 [0.44 0.89] | 0.17 |
| Hippocampus | 1.14 [0.69 1.60] | 0.73 [0.53 0.94] | 0.12 |
| Middle temporal | 0.88 [0.55 1.21] | 0.77 [0.61 0.93] | 0.54 |
| Parahippocampal | 1.15 [0.73 1.56] | 0.67 [0.53 0.81] | **0.043** |
| Fusiform | 0.85 [0.59 1.12] | 0.60 [0.49 0.70] | 0.087 |
| Inferior parietal | 0.64 [0.36 0.92] | 0.58 [0.45 0.71] | 0.71 |
| Isthmus cingulate | 0.59 [0.36 0.82] | 0.38 [0.28 0.47] | 0.097 |
| Whole brain | 0.52 [0.37 0.67] | 0.45 [0.35 0.54] | 0.44 |

**Table S3**

| Measure | Annual Atrophy  Rate (%)  MCI Aβ^+^Ptau^−^ (N=16) | Annual Atrophy  Rate (%)  MCI Aβ^−^Ptau^−^ (N=31) | p-val |
| --- | --- | --- | --- |
| Amygdala | 1.38 [0.61 2.16] | 1.20 [0.74 1.65] | 0.69 |
| Entorhinal | 1.36 [0.62 2.10] | 1.34 [0.93 1.74] | 0.96 |
| Hippocampus | 2.58 [1.52 3.65] | 1.45 [0.91 1.99] | 0.075 |
| Middle temporal | 1.42 [0.93 1.92] | 0.94 [0.56 1.31] | 0.14 |
| Parahippocampal | 1.16 [0.60 1.71] | 0.96 [0.60 1.33] | 0.57 |
| Fusiform | 0.95 [0.51 1.39] | 0.77 [0.49 1.05] | 0.51 |
| Inferior parietal | 0.87 [0.45 1.30] | 0.64 [0.34 0.95] | 0.4 |
| Isthmus cingulate | 0.83 [0.50 1.15] | 0.56 [0.33 0.80] | 0.21 |
| Whole brain | 0.71 [0.44 0.97] | 0.66 [0.40 0.91] | 0.79 |

**Table S4**

| Measure | Annual Atrophy  Rate (%)  MCI Aβ^+^Ptau^+^ (N=111) | Annual Atrophy  Rate (%)  MCI Aβ^−^Ptau^−^ (N=31) | p-val |
| --- | --- | --- | --- |
| Amygdala | 2.91 [2.59 3.23] | 1.20 [0.74 1.65] | **<10^-6^** |
| Entorhinal | 2.62 [2.36 2.87] | 1.34 [0.93 1.74] | **3x10^-6^** |
| Hippocampus | 2.38 [2.10 2.66] | 1.45 [0.91 1.99] | **0.004** |
| Middle temporal | 2.15 [1.86 2.43] | 0.94 [0.56 1.31] | **4x10^-6^** |
| Parahippocampal | 1.98 [1.76 2.20] | 0.96 [0.60 1.33] | **2x10^-5^** |
| Fusiform | 1.78 [1.58 1.98] | 0.77 [0.49 1.05] | **<10^-6^** |
| Inferior parietal | 1.58 [1.35 1.81] | 0.64 [0.34 0.95] | **9x10^-6^** |
| Isthmus cingulate | 1.24 [1.06 1.42] | 0.56 [0.33 0.80] | **3x10^-5^** |
| Whole brain | 0.95 [0.83 1.07] | 0.66 [0.40 0.91] | **0.048** |

ADNI

Data used in the preparation of this article were obtained from the Alzheimer’s Disease Neuroimaging Initiative (ADNI) database (adni.loni.ucla.edu). The ADNI was launched in 2003 by the National Institute on Aging (NIA), the National Institute of Biomedical Imaging and Bioengineering (NIBIB), the Food and Drug Administration (FDA), private pharmaceutical companies and non-profit organizations, as a $60 million, 5-year public- private partnership. The primary goal of ADNI has been to test whether serial magnetic resonance imaging (MRI), positron emission tomography (PET), other biological markers, and clinical and neuropsychological assessment can be combined to measure the progression of mild cognitive impairment (MCI) and early Alzheimer’s disease (AD). Determination of sensitive and specific markers of very early AD progression is intended to aid researchers and clinicians to develop new treatments and monitor their effectiveness, as well as lessen the time and cost of clinical trials.

The Principal Investigator of this initiative is Michael W. Weiner, MD, VA Medical Center and University of California – San Francisco. ADNI is the result of efforts of many co- investigators from a broad range of academic institutions and private corporations, and subjects have been recruited from over 50 sites across the U.S. and Canada. The initial goal of ADNI was to recruit 800 adults, ages 55 to 90, to participate in the research, approximately 200 cognitively normal older individuals to be followed for 3 years, 400 people with MCI to be followed for 3 years and 200 people with early AD to be followed for 2 years. For up-to-date information, see www.adni-info.org.

PARTICIPANTS

The ADNI general eligibility criteria have been described elsewhere (Petersen RC, Aisen PS, Beckett LA, Donohue MC, Gamst AC, Harvey DJ, et al. Alzheimer's Disease Neuroimaging Initiative (ADNI): clinical characterization. Neurology. 2010; **74**(3): 201-9). Briefly, participants are not depressed, have a modified Hachinski score of 4 or less, and have a study partner able to provide an independent evaluation of functioning. HC participants have a Clinical Dementia Rating (CDR) of 0. Participants with MCI have a subjective memory complaint, objective memory loss measured by education-adjusted scores on Wechsler Memory Scale Logical Memory II, a CDR of 0.5, preserved activities of daily living, and absence of dementia. Participants with AD have a CDR of 0.5 or 1.0 and meet National Institute of Neurological Disorders and Stroke and Alzheimer's Disease and Related Disorders Association criteria for probable AD.
